# Different exercise therapies for treating heart failure: A protocol for overview of systematic reviews and network meta-analysis

**DOI:** 10.1097/MD.0000000000022710

**Published:** 2020-10-16

**Authors:** Li Zhang, Xiao-Peng Zhao, Li-juan Qiao, Wan-xia Wei, Min Wei, Jin Ding, Ying-dong Li

**Affiliations:** a The Third Ward of Cardiovascular Clinical Medical Center, Affiliated Hospital of Gansu University of Chinese Medicine; b Evidence-Based Nursing Center, School of Nursing, Lanzhou University; c General Department of Children, Gansu provincial Maternity and Child-Care Hospital; d Gansu University of Chinese Medicine, Lanzhou, China.

**Keywords:** exercise therapy, heart failure, meta-analysis, systematic review

## Abstract

**Background:**

Exercise therapies has been shown to be safe and effective as a non-pharmacological management for treating heart failure, At the same time, many clinical trials, systematic review, and meta-analyses have demonstrated the advantages of exercise therapies in heart failure. However, the methodological quality of these systematic reviews and the differences in efficacy between different exercise modes are unclear. Therefore, this study intends to overview of systematic reviews and network meta-analysis of exercise therapies intervention in heart failure, and finally to rank the effects of exercise therapies in the intervention of heart failure, so as to provide certain reference for clinical decision-making.

**Methods:**

From the seven databases: PubMed, EMBASE.com, Web of Science, the Cochrane Library, Chinese biomedical literature database (CBM), Chinese National Knowledge Infrastructure (CNKI), Wan fang Database, and Chongqing VIP (CQVIP) databases. To search for systematic or meta-analysis of different exercise therapies for heart failure from inception to August 2020. According to the inclusion criteria and exclusion criteria, the two researchers independently selected articles and extracted data. In case of differences, a third party shall be sought for settlement. The AMSTAR2 scale, PRISMA scale and GRADE were used to assess the quality and evidence grade of the literature. The eligible randomized controlled trials (RCTs) were selected from the included systematic reviews and updated RCTs from the above systematic reviews to August 2020. GRADE was used for the risk of bias of the included RCTs. Pairwise meta-analyses were performed using the random-effects model, and network meta-analysis of the included RCTs were performed the frequentist framework. All data analyses were completed in Stata 15.0.

**Results:**

Finally, a total of 33 articles related to systematic review and meta-analysis were included, there are 28 articles in Chinese and 5 articles in English. The results of this overview and network meta-analysis will be submitted to a peer-reviewed journal for publication.

**Conclusion:**

This review will provide a comprehensive overview of existing systematic reviews of exercise therapies interventions for heart failure and provide recommendations for clinical practice or guidelines.

**Protocol Registration:**

INPLASY202080118.

## 1 Introduction

Heart failure (HF) is a complex clinical syndrome caused by impaired ventricular structure or function resulting in fullness and ejection dysfunction, It‘s clinical manifestations include dyspnea, fatigue, and fluid retention, the first of which leads to limited mobility, and the second leads to congestion of the lungs and/or internal organs and/or peripheral edema,^[1]^ which severely affects the patient's quality of life. It is estimated that the current prevalence of HF in the world is 38 million, the prevalence of HF in adults in Europe is 1% to 2%, and in China it has increased to 2% to 3%. The mortality and rehospitalization rates are also high.^[2–5]^

The treatment goal of Chronic Heart Failure (CHF) is to improve cardiac function and eliminate symptoms, improve quality of life and survival. Although clinical drug therapies under the guidance of the latest guidelines can alleviate the clinical symptoms and improve the survival rate of patients with HF, it still needs to be combined with non-drug management for better intervention effect.^[6]^ Exercise rehabilitation plays an increasingly important role in the non-drug management of HF. It has been proved to be safe and effective and can improve patients’ cardiac function, increase their exercise endurance and improve their quality of life, which has been recommended by various guidelines.^[6–8]^

Systematic review (SR) is a high-level evidence type, which is one of the important basis for clinical decision-making, and has important reference value in the development of clinical practice guideline.^[9,10]^ It can also provide information on the level of healthcare management and policy making by providing research-based answers to important questions about health systems.^[11]^ Overview of SR is a comprehensive research method to comprehensively collect the relevant SR of the treatment and diagnosis of the same disease or the same health problem for overview, which can provide more concentrated and high-quality evidence for the users of evidence.^[12]^ It has also important reference value for the formulation of clinical guidelines, but poor quality SR can also mislead decision makers.^[13]^ In recent years, many clinical trials, SR and meta-analysis have proved the advantages of exercise therapies in HF. However, due to the limitations of methodological quality and reporting quality of individual studies, the level of evidence will be affected to some extent. And because of the variety of exercises involved, whether exercise therapies can be used as a complementary alternative to HF for clinicians, and which exercise should be selected first in clinical practice, lack of high-quality evidence support, and there is no uniform standard. Finally, the effectiveness and safety of clinical interventions will be affected.

Therefore, this study intends to overview of SRs and network meta-analysis of exercise therapies intervention in HF. The Assessment of Multiple Systematic Reviews-2 (AMSTAR2) scale^[14–18]^ will use to evaluate the methodological quality of the included literature. The (PRISMA) statement^[19,20]^ will use to evaluate the report quality of the literature. The GRADE system^[21,22]^ will use to evaluate the quality of evidence for the results of the included studies. In addition, Network Meta-analysis will use to rank the effects of exercise therapies on HF, so as to provide some references for clinical decision-making.

## 2 Methods

### 2.1 Study registration

This protocol has been registered at INPLASY202080118. This study will follow the guidelines of the Preferred Reporting Items for SR and Meta-Analysis Protocol statement.^[23,24]^

### 2.2 Data sources and search strategy

This study will search the following databases: PubMed, Web of Science, Cochrane Library, EMBASE, Chinese biomedical literature database (CBM), China National Knowledge Infrastructure (CNKI), Chongqing VIP (CQVIP), and Wan Fang database. Related words will be searched by medical subject headings (Mesh) or text word search. The retrieval strategy will be designed for each database. The language is limited to English and Chinese, and there are no other restrictions. The search strategy of PubMed and EMBASE are shown in Tables 1 and 2. When the screening is completed, check the references list of included SR and meta-analysis to determine whether other SR and meta-analysis can be included.

**Table 1:**
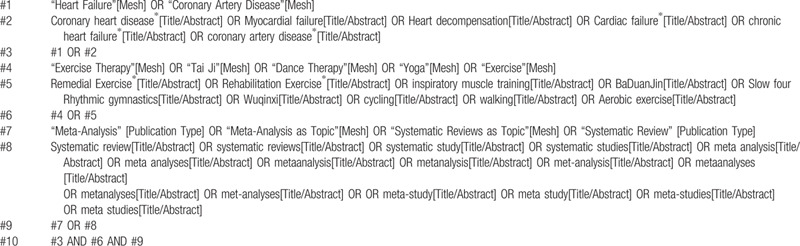
A draft search strategy using PubMed.

**Table 2:**
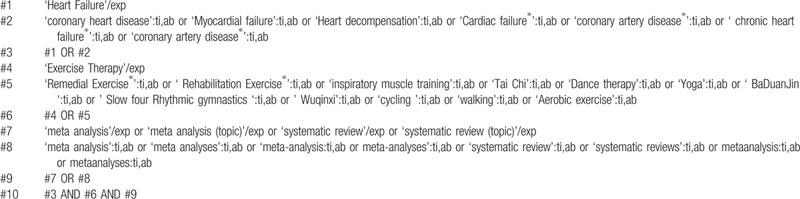
A draft search strategy using Embase.

### 2.3 Eligibility criteria for study selection

#### 2.3.1 Types of study

This study will include SR or meta-analysis in which exercise therapies was used for the treatment of patients with HF. The SR must be in English or Chinese, and up to standard of the following subjects, interventions, and outcome indicators.

#### 2.3.2 Types of participants

According to World Health Organization^[25]^ and “Guidelines for the Diagnosis and Treatment of Heart Failure in China,”^[26]^ this study will include participants who were diagnosed as HF, irrespective sex, age, and duration.

#### 2.3.3 Types of interventions

All exercise therapies interventions for HF are included, such as Aerobic exercise, inspiratory muscle training (IMT), Qigong, Tai Chi, Dance, Yoga, and so on.

#### 2.3.4 Types of outcome measures

The primary outcomes were peak oxygen uptake, left ventricular ejection fraction (LVEF), left ventricular end-diastolic diameter (LVEDD), ventilation over carbon dioxide slope, oxygen uptake efficiency slope, exercise oscillatory ventilation, rest and peak pulmonary end-tidal CO_2_. The secondary outcomes were blood pressure, heart rate, 6-minute walk test (6MWT), brain natriuretic peptide (BNP), and quality of life (QoL).

#### 2.3.5 Exclusion criteria

1. Literatures published repeatedly by the same author or with duplicate data;
2. The quality evaluation of SR or methodological research literatures;
3. Non-Chinese and English literatures;
4. The full text of the literature is not available.

### 2.4 Data collection and analysis

#### 2.4.1 Selection of studies

**Figure 1:**
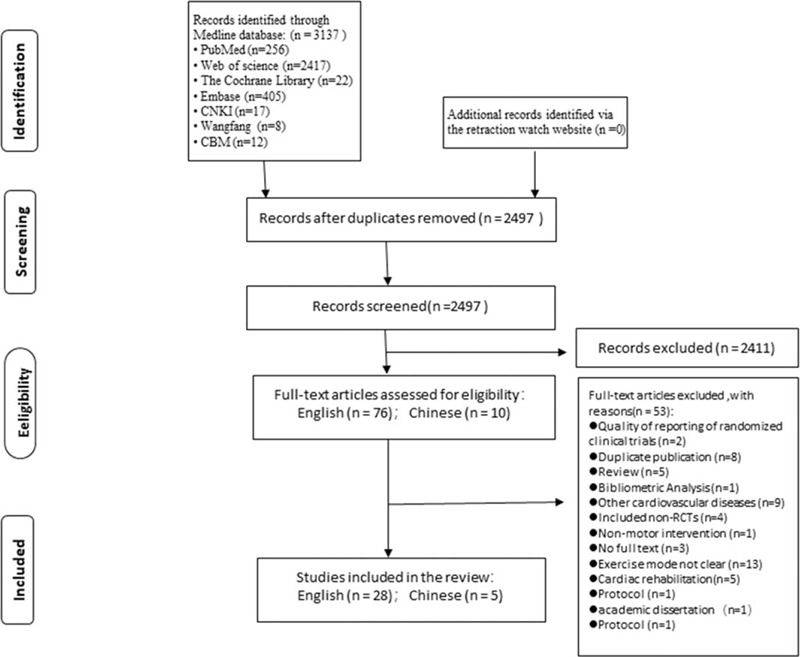
The flowchart of the screening process.

Two independent reviewers (LZ and LJQ) will check titles/abstracts of all potential literatures, and will remove irrelevant studies. We will read full-text of potential trials to determine if they fulfill all inclusion criteria. Excluded studies will be listed with specific reasons in a table. Any divergences will be solved by consulting a third reviewer (WXW). The procedure of study selection is demonstrated in a flow chart (Fig. 1).

#### 2.4.2 Data extraction and management

Two researchers (MW and WXW) applied EndNote X 8.0 (Thomson Reuters (Scientific) LLC Philadelphia, PA) Software manages literature. The pre-designed Microsoft Excel 2019 software table will be used to extract the following information: author, year of publication, number of references, number of cases, interventions, control measures, method quality assessment tool, safety assessment, and risk of bias.

#### 2.4.3 Quality appraisal

The AMSTAR-2 measurement tool was used for methodological evaluation, it uses items 2, 4, 7, 9, 11, 13, and 15 as key items affecting the SR production and the validity of its results, and the remaining items as non-key items.^[27]^ If ≤1 non-critical item is not satisfied, it will be evaluated as “high” quality level. If >1 non-key items is not sufficient, the price is rated as “medium” quality level; if a key item is not satisfied, with or without a non-key item, the quality level is “low” level. If >1 key item is not satisfied, with or without non-key item, it will be evaluated as “very low” quality level.^[28]^

The (PRISMA) statement was used for reporting quality evaluation,^[27]^ it consists of 27 items. The scoring principle is 1 point for complete report, 0.5 point for partial report and 0 point for non-report. The full score is 27 points, if ≤15 means relatively serious information defect, 15 to 21 means certain defect, 21 to 27 means relatively complete report.

The GRADE tool was used to grade the evidence quality of the major outcome indicators. In the GRADE evaluation,^[22]^ there was no evidence of downgrade as high quality, 1 item is downgraded to medium quality, 2 items are downgraded to low quality, and 3 items or more are downgraded to very low quality.

### 2.5 Data synthesis

First, pairwise direct comparisons will be performed, and then network meta-analysis will use to analyze the two-arm studies in which the three-arm studies will divide into pairwise comparisons. For dichotomous variables, odds ratios (ODD Radio, OR) and 95% confidence intervals (CI) will be used to evaluate the effectiveness of different exercise therapies in improving HF. For continuous variables, they will be expressed as standard mean difference (SMD) and 95%CI. The heterogeneity of pairwise comparison will be determined by *I*^2^ and *P*-values. If *I*^2^ ≤50% and *P* ≥ .05, there was no significant heterogeneity in the included study. Significant heterogeneity will be indicated when *I*^2^ > 50% or *P* < .05.^[29]^

We will use the random effect model of Stata.15.0 to conduct paired meta-analysis of direct evidence. By comparing direct and indirect estimates and calculating inconsistencies within each evidence closed-loop, differences between direct and indirect evidence for the same comparison are explored.^[30]^ The node-splitting method is used to evaluate whether there is inconsistency in all existing comparative analysis, that is, whether there is inconsistency in direct and indirect evidence.^[31]^ For the comparison of the effectiveness of intervention measures, the therapeutic effect of intervention measures was ranked by using the surface area under the cumulative curve and the average level as evaluation indexes. The larger the surface area under the cumulative curve and the smaller the average level were, the better the therapeutic effect of intervention measures would be.^[32]^ Comparative corrected funnel plots are used to detect potential small sample effects or publication bias.^[33]^

### 2.6 Subgroup analysis

If possible, we will do some extra subgroup analyses according to the results of heterogeneity and inconsistency (such as age, intensity of exercise). We will also investigate the sources of heterogeneity to determine the robustness and reliability of the consolidated results.

## 3 Results

### 3.1 Results of selected studies

Three thousand one hundred and thirty-seven references were initially detected, and 658 duplicates were excluded. After reading the titles and abstracts, 2411 articles were excluded that the type of research and intervention measures were not consistent with the theme. By further reading the full text, 53 references were excluded including exercise mode unclear, other cardiovascular diseases and other cardiac rehabilitation methods. Finally, 33 literatures were included for analysis. The screening details are shown in Figure 1.

### 3.2 Interventions of included studies

Among the literatures that met the requirements, the studies on the intervention of exercise therapies for HF were as follows: 14 Traditional Chinese Exercise, 12 aerobic exercise, 4 IMT, 1 yoga and 2 dance therapies.

## 4 Discussion

The current data suggest that the effectiveness of exercise therapies in cardiac rehabilitation of patients with HF has been established, but further research is needed to determine the optimal exercise pattern, exercise duration, exercise combination, and the HF population that will benefit the most. The results of our review will provide a comprehensive description on reporting and methodological quality of existing SRs/MAs and effects of exercises therapies for HF.

## Author contributions

Li Zhang, Xiao-Peng Zhao, and Ying-Dong Li conceived the study, developed the criteria. Li Zhang, Wan-Xia Wei, Li-Juan Qiao, Min Wei Searched the literature, In charge of extracting data, and verification. Li Zhang and Wan-Xia Wei analyzed the data. Li Zhang and Jin Ding wrote the protocol and revised the manuscript. Xiao-Peng Zhao provided expertise on treatments, outcomes and related knowledge. Ying-Dong Li checked all work of the review. All authors have read and approved the final manuscript.

**Conceptualization:** Li Zhang, Xiao peng Zhao, Jin Ding.

**Data curation:** Li Zhang, Li juan Qiao, Wan xia Wei, Min Wei.

**Formal analysis:** Li Zhang, Li juan Qiao, Wan xia Wei.

**Investigation:** Xiao peng Zhao, Li juan Qiao, Min Wei.

**Methodology:** Xiao peng Zhao, Jin Ding.

**Project administration:** Ying dong Li.

**Supervision:** Ying dong Li.

**Writing – original draft:** Li Zhang.

**Writing – review & editing:** Jin Ding.
